# Methadone as a “Tumor Theralgesic” against Cancer

**DOI:** 10.3389/fphar.2017.00733

**Published:** 2017-10-31

**Authors:** Marta Michalska, Arndt Katzenwadel, Philipp Wolf

**Affiliations:** Department of Urology, Medical Center – University of Freiburg, Faculty of Medicine, University of Freiburg, Freiburg, Germany

**Keywords:** methadone, cancer pain, antitumor activity, chemosensitization, tumor theralgesic

## Abstract

Methadone has beneficial characteristics as an analgesic against cancer pain, including high bioavailability, multiple receptor affinities, and lack of active metabolites that might induce adverse side effects. However, methadone has an own pharmacological profile that should be considered in the treatment of cancer patients. There is evidence from preclinical studies that methadone could also elicit antitumor activity by downregulating the threshold of apoptosis and to enhance the effects of different chemotherapeutic agents. This confirms the concept of using methadone as a chemosensitizer in the future treatment of cancer. Our article discusses major issues about the role of methadone as a possible “tumor theralgesic,” combining tumor therapeutic and analgesic activities.

## Introduction

Opioids are low-molecular-weight substances comprising (i) opiates, naturally found in the opium of the plant *Papaver somniferum* (e.g., opium, morphine, or codeine), (ii) semisynthetic opioids (e.g., heroin, hydromorphone, or buprenorphine), and (iii) synthetic opioids (e.g., fentanyl, tramadol, or methadone). They have analgesic activities and are all defined to bind to opioid receptors (OR) that are divided into the three major subclasses of mu, delta, and kappa OR (Pathan and Williams, 2012). OR are G protein-coupled receptors that have been localized in cells of the dorsal root ganglia and in their central terminals in the spinal cord dorsal horn. They are also present in the peripheral terminals of primary afferent neurons (Lamotte et al., 1976; Elde et al., 1995). Opioids and their metabolites have various effects on different OR. Generally, their analgesic activity depends on their pharmacokinetics, the OR regulation, the nature and course of pain, and the extent of inflammation (Chevlen, 2003; Coppes and Sang, 2017).

## Methadone

Methadone was first synthesized in the 1930s and is a lipophilic, basic drug (pKa 9.2) available as a hydrochlorid powder formulation. It structurally belongs to the class of diphenylpropylamines. Two enantiomers exist with dextrorotatory (S-) and levorotatory (R-) methadone, whereby R-methadone was shown to have a 10-fold higher affinity for mu1 receptors than S-methadone (Fainsinger et al., 1993; Kristensen et al., 1995). Usually, the racemic mixture of both is used for pain management.

Methadone can be orally, parenterally, or rectally given and is a potent agonist mainly at the mu1, but also on the kappa and delta OR (Kristensen et al., 1995). Methadone has also antagonistic activity at the ionotropic N-methyl-D-aspartate glutamate receptor (NMDAR). This made methadone a candidate for the clinical application against neuropathic pain syndromes. The clinical impact of NMDAR antagonism, however, has not been adequately explored (Hewitt, 2000).

The bioavailability of orally administered methadone is high and varies between 41 and 90% (Meresaar et al., 1981; Nilsson et al., 1983). Presumably due to its lipophilic properties, methadone has a high affinity to tissues, especially brain, liver, or fatty tissues, where it accumulates after multiple administrations (Sawe, 1986). A total of 60–90% of methadone was shown to form complexes with plasma proteins. Highest plasma peaks of methadone can be measured after about 4 h with a beginning of decline after about 24 h (Inturrisi and Verebely, 1972).

The elimination half-life of methadone varies widely between individuals (5–130 h) (Eap et al., 2002). These changes are mainly caused by the mentioned high tissue and plasma accumulations as well as by the methadone metabolism via cytochrome P450 (CYP) enzymes in the liver and the gut. Methadone is metabolized by N-demethylation into the two inactive metabolites 2-ethylidene-1,5-dimethyl-3,3-diphenylpyrrolidine and 2-ethyl-5-methyl-3,3-diphenylpyraline by CYP3A4, which expression varies widely among individuals and ethnic populations (Oda and Kharasch, 2001; Ferrari et al., 2004; Paine et al., 2006; McGraw and Waller, 2012). Additionally, methadone can be metabolized by CYP2D6, for which genetic differences between ethnic populations were also described (Wu et al., 1993; Begre et al., 2002). In the context of the hepatic metabolism of methadone, possible interactions with other drugs have to be noted. Especially drugs, like antidepressants, anticonvulsants, benzodiazepines, macrolide antibiotics, or antifungals can lead to an altered metabolism and adverse side effects, since these drugs are inhibitors, inducers, or substrates of CYP3A4 or CYP2D6 (Fishman et al., 2002; Ferrari et al., 2004; Weschules et al., 2008). The pharmacokinetics and pharmacodynamics of methadone can also be affected to a certain degree by P-glycoprotein (Pgp), which acts as an efflux transporter for a huge variety of lipophilic drugs in the liver, intestine, and kidney, and at the blood–brain barrier (Tanigawara, 2000).

Methadone and its metabolites are eliminated via urinary and fecal clearance (Nilsson et al., 1982). There is evidence that in renal damage, the parent compound and the metabolites are mainly excreted via feces (Kreek et al., 1980). Therefore, methadone is recommended as an analgesic for the treatment of patients with renal failure (Kreek et al., 1980; Dean, 2004).

## Methadone for the Treatment of Cancer-Related Pain

There are more than 14 million new cancer cases and 8 million deaths by this disease estimated worldwide every year (Ferlay et al., 2015). Cancer-related pain can have a negative influence on the quality of life and patients might not be able to take part in their normal daily activities. Cancer-related pain may be caused by pressing, invasion, and destruction of tissues and organs by the tumors itself. It may also be evoked by tumor diagnosis or treatment procedures, e.g., by surgery, chemotherapy, or radiation. A recently published meta-analysis revealed that pain prevalence rates were 39.9% after curative treatment, 55.0% during treatment, and 66.4% in advanced, metastatic, or terminal stages. Moderate to severe pain (Numeric Rating Scale (NRS) ≥ 5) was reported by 38.0% of the patients (van den Beuken-van Everdingen et al., 2016).

Cancer-related pain can be relieved by opioids according to the WHO pain ladder (Nersesyan and Slavin, 2007). In a recent review enclosing six different studies with 388 participants, the effects of methadone on cancer pain were analyzed. Generally, pain was reduced from moderate or severe to mild or no pain in most cases and no difference in comparison with the analgesic effects of morphine was identified. Adverse events were mainly sleepiness, constipation, and dry mouth. Withdrawals and deaths with methadone were uncommon apart from one study, where many patients died, irrespectively of treatment group (Nicholson et al., 2017). In a recent study, methadone was also administered to cancer patients suffering from the malignant psoas syndrome (MPS). MPS is a relative rare syndrome, in which the iliopsoas muscle in the lower lumbar region is impaired by the tumor and pain caused by MPS is generally difficult to control. Methadone was able to improve the NRS to assess patients’ pain levels (0–10) by average of -7.3 points (95% CI -4.97, -9.69) and patients were able to move without assistance. The average time until symptom improvement was 2.3 days after methadone treatment (95% CI 1.86, 2.80). Based on these observations, methadone is considered as a valuable therapeutic agent for MPS patients (Takase et al., 2015).

In a recent study, the use of methadone as a first-line opioid for cancer patients was examined. In the retrospective study, methadone caused a considerable decrease of cancer pain, which was comparable to other strong opioids used. Remarkably, patients receiving methadone had to rotate to other opioids less frequently than patients treated with other opioids (15 versus 50%; *p* < 0.001) and time to rotation was shown to be significantly longer (20.6 ± 4.4 versus 9.0 ± 2.7 days; *p* < 0.001) (Peirano et al., 2016).

Usually, methadone is preferably applied for opioid rotation and administered to patients with cancer pain, when insufficient analgesia with other opioids and/or difficult to treat adverse side effects appear (de Stoutz et al., 1995; Mercadante et al., 2001, 2005; Zimmermann et al., 2005). In a prospective open-label study with 145 cancer patients, methadone as a second-line opioid was found to be efficacious and safe. The opioid was able to decrease the median worst pain score from 9 (IQR: 8–10) to 6 (IQR: 3–8), and the median average pain score from 6 (IQR: 5–7) to 4 (IQR: 2–5) at day 28 after rotation (*p* < 0.0001) without increase of opioid toxicity (Porta-Sales et al., 2016).

An systematic analysis examining opioid conversion ratios with methadone comprised 41 clinical studies and case reports, in which most of the enclosed patients had cancer (*n* = 625; 88.9%). Rotations were successful in 46–89% of all cases. Nevertheless, a superiority of one method of rotation over another could not be identified due to size limited studies and inhomogeneous patient collectives. Comparability was also limited due to different administration routes, observation periods, and outcome measurements as well as involvement of drugs influencing the metabolism and analgesic effects (Weschules and Bain, 2008). Similarly, in an analysis including 15 retrospective and 10 prospective studies, success rates between 71.7% and 93.0% for different methods of rotation to methadone were described. Based on low evidence, only a trend was identified to avoid adverse side effects by applying methadone in a three-day switch with increasing methadone doses. The authors recommended further studies to identify most suitable rotation procedures and to clinically monitor the patients in a close setting during the first days of rotation to avoid adverse side effects (McLean and Twomey, 2015). It is therefore recommended to individualize equianalgesic dose ratios in view of pain level and possible adverse side effects. Furthermore, comorbidities and contraindicated drugs that might influence the metabolism and effectivity of methadone need to be taken into consideration (Mercadante and Bruera, 2006).

Drowsiness, confusion, miosis, nausea, constipation, antidiuresis, hypotension, and exacerbation of asthma are possible adverse side effects of methadone (Nicholson et al., 2017). High doses of methadone were also linked with a life-threatening prolongation of the QT interval resulting in a potentially fatal arrhythmia, called torsade de points (Krantz et al., 2002; Kornick et al., 2003; Cruciani et al., 2005; Chugh et al., 2008). It is presumed that methadone inhibits potassium channels required for rapid cardiac muscle repolarization (Lin et al., 2009). Therefore, it is recommended to perform ECG examination at the time of methadone initiation and dose escalation and when patients (i) additionally take drugs that are substrates of the CYP3A4 or CYP2D26 enzymes or block the human Ether-a-go-go-Related Gene (hERG) protein in the potassium channel of cardiac tissue or (ii) have risk factors for prolonged QT interval (Kornick et al., 2003; Cruciani et al., 2005).

## Antitumor Activities of Methadone

Besides expression in the brain and spinal cord, mu, kappa, and delta OR were also described in different tumor cell lines and tumor tissues (e.g., breast, lung, esophagus, stomach, and prostate) and associated with staging, grading, metastatic spread, and survival (Zhang et al., 2013; Zylla et al., 2013; Singleton et al., 2014; Yao et al., 2015; Connolly et al., 2017).

The observation that OR are expressed in tumor tissues led to the rationale that methadone could also act on tumor cells. Maneckjee and Minna (1992) found that methadone significantly inhibited the *in vitro* and *in vivo* growth of human lung cancer cells. They showed that methadone concentrations in the nanomolar range changed the cell morphology and viability irreversibly after 24 h exposure. The inhibitory effects after 120 h were marked by a decrease of cAMP and inhibition experiments with the OR antagonist naltrexone suggested the requirement of OR for methadone induced pathways (Maneckjee and Minna, 1992). In small cell lung carcinoma (SCLC) cells, apoptosis was identified as the mechanism for growth inhibition by methadone. The apoptotic pathways were marked by increased levels of mitogen-activated protein (MAP) kinase phosphatases and inactivation of MAP kinase, followed by a downregulation of the anti-apoptotic Bcl-2 protein. Interestingly, cell response to methadone was impaired by the addition of bombensin, an autocrine growth-stimulatory factor that plays a critical role in early events of pulmonary carcinogenesis, and cells secreting bombensin were shown to be methadone resistant (Heusch and Maneckjee, 1999).

Since methadone was shown to lower the threshold for the induction of the intrinsic apoptotic pathway, the drug was combined with other apoptosis-inducing agents for enhanced efficacy. Singh et al. (2011) combined methadone with the BH3 mimetic ABT-737, which is known to selectively inhibit anti-apoptotic members of the Bcl-2 family (Bcl-2, Bcl-w, and Bcl-xl), but not Mcl-1. They demonstrated that both substances acted synergistically on cell lines established from refractory childhood leukemia based on decreasing Mcl-1 levels evoked by methadone. The Mcl-1 loss already occurred after 6 h of treatment, suggesting a mechanism different from transcriptional modulation. Since the proteasomal inhibitor bortezomib blocked the effect of methadone, proteasomal degradation of Mcl-1 was proposed as the methadone’s mode of action (Singh et al., 2011).

Methadone was also shown to act as a chemosensitizer in leukemia cells. Whereas methadone concentrations up to 10 μg/ml led to an induction of apoptosis in about 5–40% of the cells after 120 h, the combination with doxorubicin resulted in up to 95% apoptosis. A correlation between the OR expression and the response to methadone was found. Mechanistically, the influence of methadone in combination with doxorubicin on the intrinsic apoptotic pathway was characterized by an enhanced downregulation of Bcl-xl and XIAP as well as effector caspase activation and poly-(ADP-ribose) polymerase (PARP) cleavage. The combination treatment also led to a significant inhibition of tumor growth in a mouse xenograft model (Friesen et al., 2013). Comparable results were obtained in glioblastoma cells (Friesen et al., 2014).

The role of methadone as an enhancer of apoptosis has only been demonstrated in the laboratory yet. Future clinical trials are necessary to confirm the preclinical results. In a first retrospective analysis, the safety and tolerance of 20–35 mg methadone per day in 27 glioma patients treated with radiochemotherapy, chemotherapy, or anti-angiogenetic substances, such as bevacizumab, were evaluated. A total of 13 patients reported adverse side effects (CTC grade 1–3 nausea, fatique, anxiety, drowsiness, obstipation, sweating, and pruritus) within the first 4 weeks with increasing methadone doses. After reaching the maximal methadone dosage, the adverse side effects remained in four patients with mild to moderate obstipation (CTC grade 2–3; *n* = 3) and nausea (CTC grade 2; *n* = 1). The comparison of the progression free survival at 6 months (PFS-6) between the intervention group and a historic control group was not significant; however, mean overall survival was not reached (Onken et al., 2017). This study gives a hint that methadone can be safely combined with standard tumor treatment. However, prospective randomized clinical trials are needed with respect to individual metabolism and tolerability.

## Conclusion

Methadone has many advantages in the management of cancer pain including low costs, high oral bioavailability, rapid onset of the analgesic effect, long half-life, and a lack of active metabolites, which can be beneficial for patients with renal failure. Moreover, it seems to be particularly suitable as a first- or second-line opioid or in difficult pain control scenarios. Recent studies show evidence that methadone can be safely given. However, further studies are needed to evaluate optimal dosing strategies, inter- and intra-individual differences in methadone metabolism, and possible drug interactions.

Different preclinical studies demonstrated that methadone can also act as an enhancer of apoptosis in cancer cells of different origin. This opens a window for its future use as an anticancer therapeutic. The effect of methadone on different cancer cells varies and seems to be dependent on the OR expression, apoptosis regulation, and the response to different chemotherapeutic agents used. Since standard tumor therapies, like radiation or chemotherapy, are mainly based on the induction of apoptosis, a rationale exists to combine them with methadone for an enhanced antitumor activity with simultaneous cancer pain relief. This would make methadone to a “tumor theralgesic,” a term created by us to describe a substance, which possesses tumor therapeutic as well as analgesic activities.

## Author Contributions

MM and AK did literature research, gave conceptual advice and critically revised the manuscript. PW designed and wrote the manuscript. All authors approved the final version of the manuscript to be published.

## Conflict of Interest Statement

The authors declare that the research was conducted in the absence of any commercial or financial relationships that could be construed as a potential conflict of interest. The reviewer HQ and handling Editor declared their shared affiliation.

## Abbreviations

Bcl-2: B-cell lymphoma 2
Bcl-xl: B-cell lymphoma-extra-large
cAMP: cyclic adenosine monophosphate
hERG: human Ether-a-go-go-Related Gene protein
Mcl-1: induced myeloid leukemia cell differentiation protein
MPS: malignant psoas syndrome
NMDA: N-methyl-D-aspartate
NMDAR: N-methyl-D-aspartate glutamate receptor
OR: opioid receptors
PARP: poly-(ADP-ribose) polymerase
XIAP: X-linked inhibitor of apoptosis protein.
